# Presenteeism in Nurses: Prevalence, Consequences, and Causes From the Perspectives of Nurses and Chief Nurses

**DOI:** 10.3389/fpsyt.2020.584040

**Published:** 2021-01-08

**Authors:** Geyan Shan, Shengnan Wang, Wei Wang, Shujie Guo, Yongxin Li

**Affiliations:** ^1^Institute of Psychology and Behaviour, Henan University, Kaifeng, China; ^2^Nursing Department, Henan Province People's Hospital, Zhengzhou, China

**Keywords:** presenteeism, nurses, chief nurses, prevalence, socio-economic cost

## Abstract

Presenteeism refers to the behavior of people who turn up for work despite complaints of ill health that should prompt rest and absence from work. The high incidence of presenteeism in the nurse population has been extensively investigated using self-reported methods to explore its effects on individual outcomes. However, few studies have examined nurse presenteeism using an “other's” perspective to verify self-reported information. Our aim in this study was to evaluate the prevalence, consequences, and causes of presenteeism in Chinese nurses from the perspectives of nurses and chief nurses. A sample of 481 nurses and 282 chief nurses from five hospitals in Henan Province, China, took part in this cross-sectional study. Participants completed the Sickness Presenteeism Questionnaire, Social Productivity Loss Questionnaire, and Causes of Nurse Presenteeism Questionnaire. The human capital method was used to estimate the monetary loss because of nurse presenteeism. We found that 94.25 and 82.08% of nurses experienced presenteeism in the past 6 months from the perspective of nurses and chief nurses, respectively. The annual monetary loss was estimated to be ¥4.38 billion and ¥2.88 billion based on the presenteeism reports from nurses and chief nurses, respectively. Workload, leave system, and conscientiousness are the main reasons for nurse presenteeism, and financial need is another important reason that is likely overlooked by chief nurses. This study provides a foundation for future research by presenting new knowledge about the prevalence, consequences, and causes of presenteeism in Chinese nurses. The findings emphasize the need for nursing managers and nursing departments to establish policy systems around paid sick leave, workload, and communication with managers to reduce nurse presenteeism and the subsequent socio-economic financial losses.

## Introduction

Presenteeism refers to the behavior of people who still turn up at their jobs despite complaints of ill health that should prompt rest and absence from work (1). Because of heavy workloads, shift work, and irreplaceable duties, nurses tend to continue working despite feeling unwell (2). Many studies in Western countries have demonstrated that there is a high incidence of presenteeism in nursing. For example, the incidence of presenteeism among registered nurses and assistant nurses in Swedish hospitals was 49 and 47%, respectively (1). Similarly, the incidence among Dutch nurses reached 50% (3). In recent years, the incidence of presenteeism has been increasing. Specifically, 52.6% of American nurses reported that they struggled to concentrate at work more than once in the preceding 4 weeks (4), and 59% of pediatric resident physicians in Canada had gone to work while sick (5). Presenteeism among nurses has attracted great attention from Chinese scholars (6–9); however, studies that focus on the prevalence of presenteeism among nurses are needed, which could serve as a basis for intervention policies.

Because of a series of negative effects on individual physical and mental health, job performance, and work productivity (10, 11), presenteeism has aroused extensive attention among scholars in China and abroad. Empirical studies have shown that presenteeism not only slows down an individual's recovery from sickness but also increases their risk of poorer health (12, 13). Presenteeism is currently associated with reduced job satisfaction, high job burnout, and poor work performance (14). Furthermore, presenteeism can generate a loss of workplace productivity (15) that translates into economic costs. For instance, some scholars estimate that the average annual financial loss from nurses' presenteeism in North Carolina is approximately 2–13 billion dollars (16). Although an increasing number of Chinese studies have focused on the relationship between presenteeism and individual outcomes (17), reports about social losses caused by presenteeism among nurses are scarce. The human capital method (18) is a widely used method to estimate monetary loss based on perceived decreased work efficiency and average salary. Utilizing this method, we would be able to estimate the financial losses as a consequence of nurse presenteeism.

It is well known that the ability of nurses to master professional health knowledge is a basic component of their core nursing competencies (19). As health care professionals, nurses are promoters of human health, caregivers, and advocates of health knowledge based on science. Therefore, they should have greater health literacy, be more attentive toward staying healthy, and be more effective at coping with health problems. Viewed through this lens, nurses should know when to rest or to ask for leave when they feel sick to recover from their illness faster. However, there is a sharp contrast between nurses' health literacy and unhealthy work behaviors (i.e., presenteeism). Some empirical studies have indicated that nurses have higher health literacy than the general population (20, 21) and that there is a higher incidence of presenteeism among nurses than among other industries (2). Therefore, it is important to try and understand why nurses insist on working when they feel ill.

The analysis of a considerable amount of literature highlights that self-report was the primary method used to investigate and to collect evidence in research on presenteeism. Despite self-report being the preferred method of measurement in many studies, it has been criticized for common source bias (22, 23). This includes ignoring the role of other important individuals in the nursing workplace, limiting data to the individual level, and data that cannot be verified with multilevel information. Therefore, how to recognize presenteeism from a new perspective has been a largely underexplored domain. Considering that chief nurses work in the front lines of nursing and are promoted from the ranks of general nurses, they have a sophisticated understanding of nursing behaviors and concepts. Additionally, as the direct leader of general nurses and voice for professional nurses (24), chief nurses not only shoulder the responsibilities of being supervisors but also engage in daily nursing work, helping nurses with their professional development, and cultivating excellent nursing staff. Their awareness of presenteeism is critical for nurses' occupational health and nursing personnel management.

Furthermore, previous literature has shown that organizational context plays an important role in presenteeism, and a supportive work climate tends to reduce the tendency of presenteeism (25, 26). For example, Wang et al. (46) found that the contextual factor leader-member exchange was positively related to presenteeism by increasing employees' approach motivation of presenteeism. Zhou et al. (47) conducted a longitudinal study and showed that supervisor support had an indirect impact on subordinates' productivity losses related to presenteeism by reducing role ambiguity. Furthermore, Mach et al. (43) indicated that supervisor support could moderate the relationship between job resources and presenteeism, which means that direct managers' support could buffer the interplay between job demands and job resources among healthcare employees. Therefore, chief nurses, the resource providers in the nurses' working environment, have been closely related to the working behaviors among nurses and their attitude toward presenteeism, which also further explains the rationality in exploring the causes and consequences of nurse presenteeism. Accordingly, this research aims to shed new light on the application of other-report methods in presenteeism by evaluating nurse presenteeism from the perspective of chief nurses. Based on the above literature, our study aims to investigate the prevalence, consequences, and causes of nurse presenteeism from the perspective of nurses and chief nurses. Specifically, we aim to use self-report and other-report methods to gain a comprehensive understanding of presenteeism among Chinese nurses and to provide basic empirical data to develop interventions for nurse management and occupational health.

## Methods

### Participants

We recruited 481 nurses and 282 chief nurses from five hospitals located in Henan Province, China, through convenience sampling. Prior to the investigation, we communicated with hospital nursing management and obtained permission. To ensure the high quality of these answers, ten trained research assistants went to these five hospitals working in pairs to distribute paper questionnaires that had already been coded. Participation took place during the nonworking time of participants. It was voluntary and anonymous. Participants were asked to place completed questionnaires in a certain office, which were sorted out and brought back by the research assistants. After data cleaning, the questionnaires of 418 nurses and 240 chief nurses were included for analysis, and the response rates were 86.90 and 85.11%, respectively. In this survey, nurses were aged from 18 to 54, with an average age of 27.93 years (SD = 5.57). The nursing tenure of nurses ranged from 1 to 33 years with an average of 6.26 years (SD = 5.66). Of the 418 nurses, 34 were male, 370 were female, and 14 did not respond to gender; 202 were married, 199 were unmarried, and 17 did not respond to marital status; 96 nurses had an associate's degree or less, 319 had a bachelor's degree and above, and 3 did not respond to this question. Chief nurses were aged from 23 to 56, with an average age of 40.98 years (SD = 6.42). The tenure in management was 1–35 years with an average length of 9.10 years (SD, 7.10). The number of nurses in their department ranged from 4 to 120 with an average of 18.81 (SD, 14.81). Of the 240 participants, 3 were unmarried, 230 were married, and 7 did not respond. Regarding the technical title, 184 had the title of nurse-in-charge or nurse practitioner, 52 had the title of associate professor of nursing or professor of nursing, and 4 did not respond. The departments where nurses and chief nurses worked included internal medicine, surgery, ophthalmology, pediatrics, obstetrics and gynecology, emergency room, outpatient service, nuclear medicine branch, international medical centre, and critical care medicine.

The Ethical Review Board of the Institution of Psychology and Behavior, Henan University approved the design of this study. All participants provided oral informed consent prior to completing the questionnaire.

### Measurement

General demographic characteristics, such as gender, age, tenure, marital status, education levels, and work units were collected.

The Sickness Presenteeism Questionnaire (SPQ) was used to assess the prevalence of presenteeism among nurses (27). The SPQ consists of two items, namely, “Although you felt sick, you still forced yourself to go to work” and “Although you had physical symptoms such as a headache or backache, you still forced yourself to go to work.” Nurses were required to rate how often they had experienced presenteeism during the previous 6 months. Each item was rated on a 4-point scale (1 = never, 2 = once, 3 = 2–5 times, 4 = more than 5 times) with high scores representing more frequent instances of presenteeism. The reliability of the SPQ is well documented, with previous research reporting a Cronbach's α score from 0.85 to 0.88 (14, 27). The internal reliability coefficient in this study was 0.88. To measure whether nurses' presenteeism complied with the perspective of chief nurses, we modified the items to formulate a chief nurses' version of the SPQ, which required chief nurses to evaluate the prevalence of presenteeism among their staff nurses. Specifically, we substituted “you” and “yourself” with “your subordinates” and “themselves” in the original questionnaire. The scoring method was the same as the original SPQ, and the internal reliability consistency coefficient in this study was 0.87.

Work productivity loss caused by presenteeism among nurses was measured through two self-developed items: “If your work productivity was 100% when you felt healthy, what was your work productivity while you felt sick?” and “How many days per month, on average, do you keep working with ill health?” These items aimed to assess whether and what the decrease in work productivity was affected by presenteeism and duration of presenteeism. Similar to the measurement of the prevalence of presenteeism, the items for productivity loss were modified for chief nurses by substituting “you/your” with “your subordinates/they” as appropriate. All participants were asked to write their answer on the horizontal line below the problem. The numerical method of using a two-version questionnaire was based on the human capital method, which is a widely used methodology for assigning monetary value to lost productivity (18). The social monetary losses caused by presenteeism can be calculated by multiplying the decrease in work productivity by the duration of presenteeism and the average practical salary for the Chinese nursing industry.

The causes of nurse presenteeism were assessed with 10 self-developed items. These items were generated from our systematic review of the relevant literature. The items can be divided into three categories: personal factors (3 items), family factors (3 items), and organization factors (4 items). We also created two versions of the nurse presenteeism causes questionnaire—one for nurses and one for chief nurses—following the same procedure as with the first two questionnaires. The expert grading method was implemented before distributing the questionnaire, and the item content validity was unanimously approved by experts who are experienced in nursing practice and nursing research. All participants were asked to report their degree of agreement with each item on a 5-point Likert scale ranging from 1 (strongly disagree) to 5 (strongly agree); high scores represented more approbation of those causes.

### Statistical Analysis

Statistical Package for the Social Sciences (SPSS version 22.0) software was used to analyse the data. A chi-square test or *t-*test was used to evaluate presenteeism against demographic variables. Enumeration data were described through absolute and relative frequencies, while a descriptive statistical method was used for quantitative data.

## Results

### Presenteeism Prevalence and Demographic Differences

The responses of nurses indicated that the mean (SD) score for nurse presenteeism was 2.72 (0.92). In the previous 6 months, 394 nurses (94.25%) experienced presenteeism (i.e., the average score of two items > 1). According to the responses of chief nurses, the mean (SD) score of nurse presenteeism was 2.23 (0.91), and 197 chief nurses (82.08%) found that their subordinates experienced presenteeism (i.e., the average score of two items > 1) in the previous 6 months. Table 1 shows the distribution of nurse and chief nurse responses on the two items of the SPQ.

**Table 1:** The distribution of presenteeism scores (%).

**Items**	**Participants**	**$\overset{¯}{x}\pm \text{s}$**	**Never**	**Once**	**2-5 times**	**>5 times**	**Prevalence (%)**
1. Although you/your subordinates felt sick,	Nurse	2.68 ± 0.95	10.53%	34.45%	31.10%	23.92%	89.47%
they still forced yourself/themselves to go to work	Chief Nurse	2.20 ± 0.96	27.23%	37.45%	23.83%	11.49%	72.77%
2. Although you/your subordinates had physical symptoms such as a headache or backache,	Nurse	2.76 ± 0.99	11.00%	30.62%	29.90%	28.47%	89.00%
you/they still forced yourself/themselves to go to work	Chief Nurse	2.26 ± 0.98	23.81%	41.99%	18.18%	16.02%	76.19%

Further statistical analysis using *t*-test showed that there were significant differences in nurse presenteeism scores in terms of nurse age, tenure, and marital status (*p* < 0.05). The results are shown in Table 2.

**Table 2:** Nurse presenteeism differences for individual characteristics.

**Characteristic**	**Categories**	***n***	**Valid %**	**Mean**	***SD***	***t***	***p***
Age	30 years old and under	302	72.25%	2.65	0.90	−2.44	0.02
	More than 30 years old	116	27.75%	2.90	0.93		
Tenure	3 years and under	151	37.10%	2.44	0.89	−4.94	0.00
	More than 3 years	256	62.90%	2.90	0.90		
Gender	Male	34	8.42%	2.59	0.77	−1.819	0.07
	Female	370	91.58%	2.74	0.93		
Marital status	Unmarried	199	49.63%	2.61	0.92	−2.124	0.03
	Married	202	50.37%	2.81	0.91		
Education level	Associate's degree or less	96	23.23%	2.71	0.93	−1.119	0.955
	Bachelor's degree and above	319	76.87%	2.72	0.92		

The results demonstrated that nurse age, tenure, and marital status were closely related to their presenteeism (*t*_1_ = −2.44, *p* < 0.05; *t*_2_ = −4.94, *p* < 0.001; *t*_3_ = −2.124, *p* < 0.05, respectively), but there were no significant relationships between presenteeism and education level or gender (*t*_1_ = −1.119, *p* > 0.05; *t*_2_ = −1.819, *p* > 0.05). The prevalence of nurse presenteeism increased significantly with increased age and tenure, and married nurses preferred to work while ill more than unmarried nurses.

### Work Productivity Loss and Socio-Economic Cost Caused by Nurse Presenteeism

Of the 394 nurses who experienced presenteeism, 368 (93.40%) reported that their work productivity was reduced when they worked while sick. When working while ill, the average work efficiency was 74.08%. In other words, the average decrease in working efficiency was 25.92%. The majority of chief nurses (94.37%) believed that their subordinate's productivity would have been reduced when they felt sick but still worked. According to their estimation, the average productivity of their subordinates in the presenteeism condition was 78.99%, which means they estimated an average decrease in working efficiency of 21.01%.

The survey by the National Health Commission of China (45) reported that the total number of Chinese nurses was more than 4 million. Meanwhile, according to the reports of the 919 Nurse Care Program (28) that was implemented by the China Social Welfare Foundation (CSWF), 76.5% of nurses earned < ¥5,000 per month, and approximately 37.6% earned < ¥3,000 per month. This study estimated that the monthly salary of Chinese nurses was ¥4,000, thus yielding an average total annual compensation of ¥192 billion. In this survey, the mean number of days per month that nurses worked while sick was 2.08, as reported by nurses who experienced presenteeism and assumed that productivity would be reduced. In accordance with the Labour Law of the People's Republic of China (29), laborers work on average 20.83 days per month. Accordingly, presenteeism days among nurses accounted for 9.99% of the total workdays based on the nurses' report. According to the report of chief nurses, the mean number of days per month that their subordinates worked while sick was 1.92 (SD, 3.23), which accounted for 9.22% of the total workdays.

Based on the human capital method, the annual monetary loss of presenteeism can be calculated by multiplying the total annual compensation of nurses with the proportion of the population whose productivity declined from presenteeism, the proportion of productivity lost and the proportion of duration of presenteeism. Therefore, this study multiplied ¥192 billion with the product of 94.25 and 93.40% (i.e., the proportion of the population whose productivity declined from presenteeism) and then multiplied it by 25.92 and 9.99%. This calculation estimated that the direct monetary loss was ~¥4.38 billion annually. However, when the calculation was performed with the data from the chief nurses (¥192 billion multiplied by the product of 82.08 and 94.37%, then multiplied it by 21.01 and 9.22%), the estimated figure was ¥2.88 billion annually.

### The Causes for Presenteeism

This study listed 10 possible causes of nurse presenteeism from the perspective of organization, family, and the individual. Overall, the average approval rating was highest for organization related causes (51.44%). The approval rating of nurses for all items ranged from 21.59 to 63.64%, and the top four items with approval ratings above 50% included workload (63.64%), leave system (63.46%), conscientiousness (59.10%), and financial needs (52.04%). A detailed distribution of the approval rating of these items is shown in Table 3.

**Table 3:** Approval rating of presenteeism causes among nurses (%).

**Category**	**Items**	**Strongly disagree**	**Somewhat disagree**	**Uncertain**	**Somewhat agree**	**Strongly agree**	**Total approval rating**
Individual	1. It is out of a sense of work responsibility that people keep working when they felt sick (Conscientiousness)	5.74	16.51	18.66	32.78	26.32	59.10
	2. When people get sick, they still go to work because of his/her personal work habits (Work habits)	7.18	23.21	29.9	28.47	11.96	40.43
	3. Some people work with illness out of passion for their jobs (Passion for work)	5.74	15.42	34.22	30.84	13.73	44.57
Family	4. People keep going to work while sick because of their financial needs (Financial needs)	3.60	17.51	26.86	35.73	16.31	52.04
	5. Some people still go to work even when they felt sick on account of feeling lonely at home (Family relationships)	13.67	27.82	36.93	16.07	5.52	21.59
	6. To avoid family conflicts, some people go to work even when they feel sick (Evade conflicts)	12.23	21.34	39.57	19.18	7.67	26.85
Organization	7. Because the organization makes it difficult to ask for leave, some people choose to continue to work when they feel sick (Leave system)	4.09	12.02	20.43	43.75	19.71	63.46
	8. Because of the intensive workload, in order to complete jobs, some people choose to work when ill (Workload)	4.07	10.77	21.53	44.50	19.14	63.64
	9. To maintain a hard-working image, some people still go to work when they feel ill (The influence of leader)	10.55	23.98	36.69	24.98	4.78	29.76
	10. Since no one can be substituted to do the work among your workmates, they do not ask for leave even when they feel sick (Job substitutability)	5.54	16.63	28.92	36.39	12.53	48.92

*Total approval rating = somewhat approval rating + strongly agree rating*.

From the chief nurses' perspective, the average approval rating was highest for organization related causes (37.99%). The approval rating across all items ranged from 5.00 to 50.00%. Similar to the nurse responses, the top three items were workload (50.00%), leave system (46.44%), and conscientiousness (44.58%), whose approval rating was higher than the disapproval rating. A detailed distribution of the approval rating of these items is shown in Table 4.

**Table 4:** Approval rating of presenteeism causes among chief nurses (%).

**Category**	**Items**	**Strongly disagree**	**Somewhat disagree**	**Uncertain**	**Somewhat agree**	**Strongly agree**	**Total approval rating**
Individual	1. Out of a sense of responsibility for work, some of your subordinates kept working even when they felt sick (Conscientiousness)	20.83	22.08	12.50	28.33	16.25	44.58
	2. When a subordinate gets sick, he/she still goes to work because of his/her personal work habits (Work habits)	22.27	23.11	23.11	27.31	4.20	31.51
	3. Some people work through illness out of passion for their jobs (Passion for work)	19.33	22.27	26.47	23.53	8.40	31.93
Family	4. Your subordinates go to work sick because of his/her financial needs (Financial needs)	24.89	19.83	32.91	17.72	4.64	22.36
	5. Some people still go to work even when they felt sick because they feel lonely at home (Family relationships)	46.67	27.08	18.33	7.50	0.42	7.92
	6. To avoid family conflict, some people go to work even when they felt sick (Evade conflicts)	42.92	24.58	27.50	5.00	0.00	5.00
Organization	7. Because the organization makes it difficult to ask for leave, some people choose to continue to work when they feel sick (Leave system)	19.67	21.34	12.55	34.31	12.13	46.44
	8. Because of the large workload, in order to complete jobs, subordinates choose to work while ill (Workload)	15.83	21.25	12.92	36.25	13.75	50.00
	9. Some people still go to work despite illness to maintain a hard-working image (The influence of leader)	26.58	30.38	26.58	14.77	1.69	16.46
	10. Since no one can be substituted to do the work of subordinates, they do not ask for leave even when they feel sick (Job substitutability)	22.69	28.15	10.08	28.15	10.92	39.07

*Total approval rating = somewhat approval rating + strongly agree rating*.

## Discussion

This study indicated that the average presenteeism score was 2.72 (SD = 0.92), with a prevalence of 94.25% in Chinese nurses from the perspective of nurses. The high prevalence was corroborated by chief nurses who reported a presenteeism prevalence of 82.08% among nurses. Older age and more senior tenure are factors that represent significantly higher rates of presenteeism, which was consistent with the results of Bierla et al. (32) on age and that of Martinez and Ferreira (44) on tenure. Marital status was another demographic factor closely related to presenteeism. It could be explained that married nurses are likely to consider not only themselves but also their family, spouse, and children. For fear of affecting family income, parenting ability, and quality of life by their absence behavior, they may be more likely to work while in poor health. Moreover, the results showed that the total estimated monetary loss was ¥4.38 billion and ¥2.88 billion based on nurse reported and chief nurse reported presenteeism, which illustrates the severity of the economic losses caused by nurse presenteeism in China and the significant need for research on nurse presenteeism.

To explore the reasons underlying presenteeism among Chinese nurses, ten possible causes of nurse presenteeism were tested in this investigation, and the results showed that workload, leave system, and conscientiousness were the main causes of presenteeism reported by nurses and chief nurses.

The shortage of the nursing workforce may be the primary cause of the high workload and strict leave system in the nursing professions. A previous survey found that the number of registered nurses per 1,000 permanent residents in China was only 2.74 by the end of 2017, far from the required number of 4.7 by the CPC Central Committee and State Council in the Healthy China 2030 Plan Compendium. On the one hand, the shortage of nurses in China increased the workload of on-the-job nurses and prolonged their work hours, such that nearly half of nurses worked over 40 h a week (30), which means that nurses, to some extent, were conscripted to sacrifice their personal health to complete their work. On the other hand, the shortage of nurses made it difficult for the managers to find substitutes when one person felt ill and asked for leave (the approval rating of the lack of job substitution was 48.92 and 39.07% for nurses and chief nurses, respectively). Thus, to ensure that there are enough nurses on duty, many medical and health institutions were forced to establish strict absence management procedures, which further makes it difficult for nurses to ask for sick leave.

In this study, conscientiousness acted as the third cause of nurse presenteeism. Conscientiousness is an important psychological trait that describes individual differences in the propensity to be self-controlled, responsible to others, hardworking, orderly, and rule abiding (31). However, previous studies have shown that highly responsible workers are more likely to be presenteeists (32). It is likely that individuals with a high degree of responsibility prefer to complete jobs by themselves rather than seek help from others (33); therefore, physical discomfort does not stop them from finishing their jobs. In addition, highly conscientious individuals tend to think more about how their absence could have a negative effect on them as individuals and the organization (32). For example, they believe that their absence might damage their image in leaders and result in difficulties in the scheduling of shift work for managers and the organization.

It is interesting that financial needs were the fourth greatest reason for presenteeism reported by nurses whose total approval rating exceeded half but less than a quarter reported by chief nurses. Previous studies have supported that there is an elevated risk of presenteeism in people with high personal financial demands (34). This finding could be the reason that lower income leads to greater economic pressure and stronger financial needs. Compared with high earners, low earners will experience more economic pressure following the same absence behavior and the corresponding pecuniary loss. To obtain an ideal economic reward, they may have a more urgent desire to complete more jobs and a stronger motivation to refuse absence. Consequently, nurses are more inclined to choose presenteeism, not absenteeism, in the case of physical discomfort. Because of the income gap between chief nurses and their subordinates, chief nurses usually have higher payment than common nurses, which may lead them to underestimate the impacts of financial needs on nurses' presenteeism.

### Theoretical Implications

Compared with existing studies such as Demerouti et al. (3) and Christopher (4), this study indicated that the prevalence of nurse presenteeism was higher in China. Our results not only enriched the empirical study of presenteeism in China but also together with the results of Lu et al. (14), which demonstrated presenteeism in enterprise employees in Taiwan Province, China has a higher prevalence of presenteeism than the United Kingdom. The results imply that presenteeism may be more prevalent in Chinese nursing occupations. Given the limited evidence, it is unclear whether there is a true East-West difference in the incidence of presenteeism. More studies are needed to explore whether and why there may be an East-West difference or cultural difference in presenteeism among nurses.

Previous studies have shown that nurse presenteeism often leads to a series of negative consequences, and the majority of these studies have used the self-reported method to explore the relationship between presenteeism and its outcomes. Selected outcome variables were mainly focused on the individual, specifically individual subjective perception, such as individual psychological health, quality of care, and burnout (3, 6, 16). A limited number of studies [just as (16)] have focused on the consequences of presenteeism from a socio-economic perspective in China. This study estimated the direct economic loss of nurse presenteeism from two perspectives, which not only verified the heavy cost of Chinese nurse presenteeism but also provided a comparatively credible result for related research on presenteeism. Exploring the causes of nurse presenteeism from multiple perspectives could contribute to a more comprehensive understanding of the role of situational factors on presenteeism. It is more conducive to obtaining the most core causes of nurse presenteeism and the causes that are easily ignored from one perspective, thus contributing to future practical intervention. In addition, the slightly lower prevalence of presenteeism and socio-economic financial loss reported by chief nurses could be due to their underestimation or nurses' overestimation. The actual prevalence of presenteeism and socio-economic financial loss could exist somewhere between other-reported and self-reported results, which provided an estimated interval to reveal the true occurrence rate and financial loss numerical value.

Finally, this study extended the application of social identity theory and job demands-resources theory to a certain degree. According to social identity theory (35), with increasing age and tenure, nurses have a clearer perception of their professional responsibility, a stronger sense of loyalty to their organization, and a closer connection between individual self-identity and organizational identity. Once absent from work, pressure in terms of responsibility and emotion will increase. It can be logically explained why people with older age and more senior tenure would choose to go to work rather than ask for sick leave when they feel sick. From job demands-resources theory, job demands require sustaining physical and psychological effort that consequently leads to a certain level of physiological and/or psychological costs (36). The workload and leave system are the typical job demands for employees. The present findings showed that nurses' job demand was the main cause that affected their presenteeism. Therefore, it may be a reliable approach to reduce presenteeism and its cost by increasing individual job resources and psychological resources (37). Furthermore, more intervention programmes on presenteeism are suggested based on the theory.

### Practical Implications

Our results indicated that nurse presenteeism highly existed, and great monetary loss was caused by it. This result suggested that medical and healthcare administrative departments should pay more attention to presenteeism among nurses and take active measures to intervene it to improve nursing quality and to minimize direct monetary losses. In addition, the close relationship between job demands and presenteeism indicated that interventions for nurse presenteeism should focus on recruiting more nurses to increase their talent reserves and implementing more high-quality training for nurse professional development. This approach would reduce nurses' workload and empower management to arrange duties flexibly. At the same time, medical and health organizations should optimize their absence management procedures to ensure that nurses who are in need have rights and opportunities to ask for leave. This issue is particularly relevant during the COVID-19 pandemic (and other similar situations) when nurses are at the frontlines fighting the virus (38), as the increasing number of patients would aggravate nurses' workloads and threaten their health. In addition, considering the high infectiousness of the coronavirus, an infected nurse might become a new source to spread the disease. Thus, an effective attendance management system is required to ensure nursing quality and nurse health.

Our investigation showed that one of the main causes of presenteeism was conscientiousness, which means that some nurses would rather be present than absent when sick to avoid the negative consequences caused by sick leave. Formerly, medical and health departments together with nursing management departments have been encouraged to strive to cultivate and to improve nurse conscientiousness to enhance the quality of nursing and patient satisfaction. However, our study revealed that it is also necessary to consider the effect of too much of a good thing (i.e., when conscientiousness with work increases too much, this may indirectly lead to unhealthy consequences such as presenteeism) to prevent and to reduce the occurrence of nurse presenteeism (39). The main causes of presenteeism suggested that management departments should reassess the nursing salary system, formulate more reasonable regulations around paid sick leave requests, and offer support for nurses to take sick leave. These measures could potentially help to reduce sick leave associated economic losses for nurses, thereby reducing the occurrence of presenteeism and the socio-economic costs of presenteeism, and a better balance between the financial needs of individual and organizational expenditures can be achieved.

Despite the perception of nurses and chief nurses being consistent regarding the prevalence of nurse presenteeism, economic losses caused by presenteeism, and the main reasons for nurse presenteeism, compared to the nurses, chief nurses underestimated those three aspects. Specifically, leaders tended to underestimate the presenteeism frequency of their subordinates and related productivity loss. Similar trends were observed for the causes of presenteeism, especially with regard to financial needs. It is these differences that make leaders more inclined to judge too severely in the process of human resource management, even though it is unintentional. Therefore, this study may shed light on how leaders should manage their subordinates and coordinate work in the future, particularly when engaging in nursing management during crisis events. For example, the COVID-19 pandemic resulted in nurses being required to manage additional sources of stress (40). This result raises the question of whether the reasons and motivations for presenteeism will change and whether the differences between superiors and subordinates will be amplified. Furthermore, whether those differences will affect the implementation of the key policy and have a negative impact on nurses' health remains unclear. Solving all these questions is necessary for nursing management departments to have a timely and comprehensive understanding of what motivates nurse presenteeism.

### Limitations and Future Research

The results of our study should be considered in light of its limitations. First, participants were recruited within one province. Although Henan Province, located in the middle of China, can generally reflect the current situation and history of China and has usually been regarded as the epitome of China in population, agriculture, economy, culture and image (41), selecting nurses from one province still has difficulties with generalizing this population to all Chinese nurses. Therefore, the results need to be verified by more studies in the future. Second, the method used to estimate the economic loss caused by nurse presenteeism was rudimentary. It was constrained to take into account the interdependence of the service object, colleagues, and departments in the whole hospital (42). Accounting for these factors may lead to greater monetary loss from secondary damage associated with patients, additional expenditure of nurses, work units and their family, and the social loss of resources related to social insurance and welfare caused by nurse presenteeism. If these indirect losses were calculated, the total loss caused by nurse presenteeism may be much larger. Although it provided initial estimates of the cost of presenteeism, ¥4.38 billion reported by nurses and ¥2.88 billion reported by chief nurses could be regarded as the upper and lower limits of the estimated interval. Thus, more precise scientific estimation methods are needed in future research. Furthermore, although this study investigated the causes of presenteeism from the perspective of nurses and chief nurses, it did not investigate chief nurses' presenteeism. This finding is consistent with the suggestion of Ruhle et al. (26) that further empirical research focuses not only on the consequences of leaders' behavior and styles for presenteeism but also on the exchange between leaders and followers. In addition, future research should utilize scientific assessments and adopt a combined study design to examine the relationships and mechanism of interaction between presenteeism and its antecedents and outcomes. What is also significant both in nurse presenteeism practice and theory is to explore the social context of presenteeism and possible spill over dynamics occurring across levels and domains (25). A clear understanding of the underlying factors of presenteeism would allow for the development of scientific and robust strategies to help prevent and to reduce presenteeism among nurses. Effective interventions could contribute to improving the professional quality of nursing and promote nurses' occupational health.

## Conclusion

This study focuses on the prevalence, consequences, and causes of nurse presenteeism from the perspectives of nurses and chief nurses. The results showed that there was a high prevalence of presenteeism in Chinese nurses, which led to a large amount of monetary loss. Workload, leave system, and conscientiousness were the main reasons for nurse presenteeism reported by both nurses and chief nurses, and financial needs were another important reason in nurses' views but not in chief nurses' views. Our results contributed to enriching the literature on nurse presenteeism and job demands-resources theory. Considering that some job context factors were the primary causes of nurse presenteeism, the healthcare management was suggested to build more reasonable regulations and humanized attendance management systems for nurses to reduce presenteeism and monetary loss caused by it.

## Data Availability Statement

The raw data supporting the conclusions of this article will be made available by the authors, without undue reservation.

## Ethics Statement

The studies involving human participants were reviewed and approved by The Ethical Review Board of the Institution of Psychology and Behavior, Henan University approved the design of this study. The patients/participants provided their written informed consent to participate in this study.

## Author Contributions

YL is the principal investigator for the study, generated the idea and designed the study. GS and SW were the primary writers of the manuscript and approved all changes. GS and WW supported the data input and data analysis. SG supported the data collection. All authors were involved in developing, editing, reviewing, and providing feedback for this manuscript and have given approval of the final version to be published.

## Conflict of Interest

The authors declare that the research was conducted in the absence of any commercial or financial relationships that could be construed as a potential conflict of interest.
